# Field programmable gate array compression for large array multispeckle diffuse correlation spectroscopy

**DOI:** 10.1117/1.JBO.28.5.057001

**Published:** 2023-05-08

**Authors:** Francescopaolo Mattioli Della Rocca, Edbert J. Sie, Ryan Catoen, Francesco Marsili, Robert K. Henderson

**Affiliations:** a The University of Edinburgh, School of Engineering, Edinburgh, United Kingdom; b Meta Platforms Inc., Reality Labs Research, Menlo Park, California, United States

**Keywords:** multispeckle, diffuse correlation spectroscopy, single-photon avalanche diode, field-programmable gate array compression

## Abstract

**Significance:**

Diffuse correlation spectroscopy (DCS) is an indispensable tool for quantifying cerebral blood flow noninvasively by measuring the autocorrelation function (ACF) of the diffused light. Recently, a multispeckle DCS approach was proposed to scale up the sensitivity with the number of independent speckle measurements, leveraging the rapid development of single-photon avalanche diode (SPAD) cameras. However, the extremely high data rate from advanced SPAD cameras is beyond the data transfer rate commonly available and requires specialized high-performance computation to calculate large number of autocorrelators (ACs) for real-time measurements.

**Aim:**

We aim to demonstrate a data compression scheme in the readout field-programmable gate array (FPGA) of a large-pixel-count SPAD camera. On-FPGA, data compression should democratize SPAD cameras and streamline system integration for multispeckle DCS.

**Approach:**

We present a 192×128 SPAD array with 128 linear ACs embedded on an FPGA to calculate 12,288 ACFs in real time.

**Results:**

We achieved a signal-to-noise ratio (SNR) gain of 110 over a single-pixel DCS system and more than threefold increase in SNR with respect to the state-of-the-art multispeckle DCS.

**Conclusions:**

The FPGA-embedded autocorrelation algorithm offers a scalable data compression method to large SPAD array, which can improve the sensitivity and usability of multispeckle DCS instruments.

## 1 Introduction

Monitoring of cerebral blood flow is key to understanding brain health and neuronal activity. Diffuse correlation spectroscopy (DCS) is an optical technique capable of measuring blood flow noninvasively using coherent near-infrared light.^1^^–^^6^ Coherent light introduced from the source to the scalp through deep tissue returns a speckle pattern at the detector, which fluctuates rapidly in intensity due to movement of the tissue, including the red blood cells therein. Measurement of the temporal intensity autocorrelation function (ACF), i.e., $g_{2}(\tau )$, of the speckle yields a correlation time constant ($\tau_{c}$), which can be used to quantify the blood flow index.

By increasing the source–detector separation over the scalp, DCS can capture photons scattered from deep into the cerebral cortex. However, the increase in separation sacrifices the signal-to-noise ratio (SNR) of the return signal, which is crucial for maximizing the precision of the $g_{2}$ measurement. To increase the SNR of the $g_{2}(\tau )$ curve [defined as the ratio between mean and standard deviation (STD) of $g_{2}(\tau )$ calculated over several integration periods], a multispeckle DCS approach was proposed and demonstrated.^6^^–^^9^ In multispeckle DCS, each independent speckle field scattered from the tissue is coupled to an independent single-photon avalanche diode (SPAD) or to each pixel of the SPAD array, showing an SNR gain that scales with the square root of the number of pixels in the SPAD camera. Despite the scalability advantage offered by SPAD camera technology, the multispeckle DCS systems reported so far were limited by the small number of pixels in the SPAD array and the lack of camera-embedded processing. The latter has required external large computing resources to sustain the high frame rate of the camera and impeded scalability to higher resolutions and multiple camera systems fitted on the scalp.

State–of-the-art hardware autocorrelators (ACs) can only operate on a low number of channels, usually in the order of 10 to 100 channels, which does not allow coupling to high resolution of state-of-the-art SPAD cameras for real-time operation. The work in Ref. 10 was the first field-programmable gate array (FPGA) implementation of AC able to scale to 1032 channels interfacing to a 32×32 SPAD array, but the normalization of the per-pixel ACF was kept in software on the host PC. To achieve the SNR scaling with number of pixels, the per-pixel ACFs need to be averaged together into a single ACF also known as ensemble ACF. Since the ensemble ACF can only be performed after the normalization of each per-pixel ACF, the AC presented in Ref. 10 must stream the ACF and the raw aggregated photon count of each pixel to the host PC. This imposes a large data rate requirement, which is not compatible with the trend of increasing pixel resolution of SPAD sensors and therefore limits the potential for achieving higher SNR in real-time multispeckle DCS. To fully exploit the large number of channels in SPAD cameras, ACs can benefit from keeping the entire elaboration pipeline locally on the FPGA, including normalization and ensemble averaging and use the limited bandwidth to the host PC to stream out the SNR-scaled ensemble ACF.

Here we present a large array SPAD camera with 128 16-lag linear ACs embedded on an FPGA able to calculate 12,288 channels and output both per-pixel ACFs and compute the ensemble ACF from 192×64  pixels in real time. This represents a 12 times increase in number of channels than in prior works.^6^^,^^7^^,^^10^ We achieved an SNR gain of 110 over a single-pixel DCS system, which is more than threefold increase in the SNR gain shown in prior works.^6^^,^^7^ Although this work focuses on the FPGA-embedded processing for multispeckle DCS, our SPAD camera can operate in the multispeckle time-domain DCS with an unprecedented precision of 33 ps time-of-flight (TOF) resolution. This is beyond the precision offered by a gated approach that typically has nanosecond scale timestamp.

## 2 Materials and Methods

**Fig. 1:**
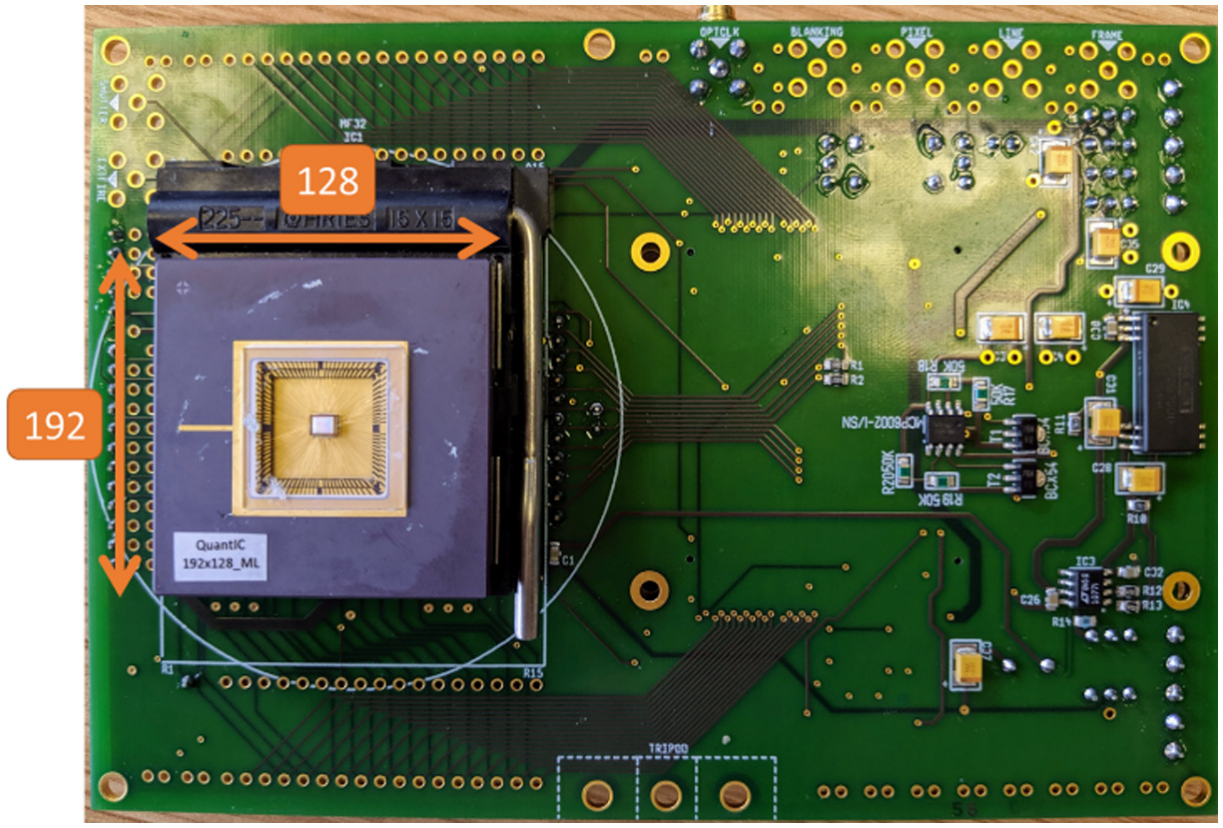
QuantICAM SPAD array circuit board used in this work.

We used a time-resolved complementary-metal-oxide semiconductor (CMOS) SPAD sensor composed of 192×128  pixels with a 33 ps TOF resolution and a 135-ns time-to-digital converter (TDC) in each pixel. The SPAD array was packaged into a camera module, referred to as QuantICAM.^11^ Part of the TDC is an 8-bit ripple counter which was repurposed to enable single-photon counting of up to 255 photons per frame exposure in each pixel. We used an Opal Kelly 7310 based on a Xilinx Artix-7 FPGA to control the sensor and to read out the chip through 64 data output I/Os. Figure 1 shows a photo of the QuantICAM SPAD array used in this work. Detailed system architecture of the QuantICAM SPAD array can be found in Ref. 11.

The sensor was read out in a rolling row readout scheme. Pairs of rows were read out in parallel from the center towards the top and bottom of the sensor. Each row was read out by 32 I/Os, each I/Os serially reading out data from 4 pixels. The maximum raw data rate from QuantICAM is 64×100  Mbit/s or 6.4  Gbit/s (800  MB/s), which is twice the data throughput of standard Universal Serial Bus (USB 3.0) connector of 3.2  Gbit/s (400  MB/s) after accounting for encoding overhead. In addition, we must perform about 400,000 autocorrelation operations (192×128  pixels, 16-lags, see Sec. 3) in each integration period (tens of ms) in real time. Such large data rates and compute-intensive operations are not sustainable with standard protocol, thus data compression on camera is needed, using FPGA, before passing the processed data to PC.

We synthesized a total of 128 ACs on the FPGA. Each AC was composed of 16 linearly spaced lags with a minimum time lag ($\tau_{\min}$) of 38.4  μs and a maximum time lag ($\tau_{\max}$) of 614.4  μs, related by $\tau_{\max}=16\;\tau_{\min}$. The sensor outputs the photon count for each $\tau_{\min}$ period, which is equivalent to the sensor exposure time. Thus the shortest $\tau_{\min}$ is limited by the maximum frame rate of the sensor of 26 kfps. Although we can, in principle, adjust $\tau_{\min}$ by increasing the exposure time forcing a lower frame rate, the longest $\tau_{\min}$ is capped by the pixel saturation value of 255 counts limited by the in-pixel counter.

Due to the limited memory resources on the chosen FPGA, only 64 columns of pixels could be stored in memory over a $g_{2}(\tau )$ integration period ($T_{\mathrm{int}}$) of 60 ms. The FPGA can therefore compute 12,288 ACFs from the group of 192×64  pixels in the SPAD array. This is half of the full array in our 192×128 SPAD array.

The ACF as a function of time lag τ for each pixel i can be estimated by computing the following equation:

$$g_{2_{i}}(\tau )=\frac{\left\langle n_{i}(t)n_{i}(t+\tau )\right\rangle }{{\left\langle n_{i}(t)\right\rangle }^{2}},$$ (1)

where n(t) is the number of photons recorded in time bin t, τ is the time lag, and the square bracket ⟨…⟩ denotes the average over the integration time $T_{\mathrm{int}}$. The denominator component is the square of the average photon count over $T_{\mathrm{int}}$. The variation in the denominator value across the time lags (16 lags) is negligible at long $T_{\mathrm{int}}$ of 60 ms. We then calculated the ensemble average of the $g_{2}$ curves measured over M pixels as

$${{\overset{¯}{g}}_{2}(\tau )|}_{M}=\frac{1}{M}\overset{M}{\underset{i=1}{\sum }}g_{2_{i}}(\tau ).$$ (2)

We measured $g_{2}$ with $T_{\mathrm{int}}=60\;\mathrm{ms}$ and repeated the measurement for up to 6 s, contiguously with no spacing in between, for a total of 100 $g_{2}$ measurements per pixel. We calculated the time average $\mathrm{MEAN}[g_{2}(\tau )]$ and $\mathrm{STD}[g_{2}(\tau )]$ of $g_{2}$ over all of the 100 integration periods. We define the SNR of the ensemble average as $\mathrm{SNR}[{\overset{¯}{g}}_{2}(\tau )]=\mathrm{MEAN}[{\overset{¯}{g}}_{2}(\tau )-1]/\mathrm{STD}[{\overset{¯}{g}}_{2}(\tau )]$.

Equation (1) was implemented on the FPGA for each pixel i with the following equation:

$$g_{2i}(\tau )=\frac{N\cdot (\sum_{k=1}^{N}n_{i}(k)n_{i}\cdot (k-f_{\tau }))}{{\left(\sum_{k=1}^{N}n_{i}(k)\right)}^{2}},$$ (3)

where k is the frame number and N is the total number of frames in an ACF integration period. The above equation must be computed for each of the $f_{\tau }=1$ to 16 frame lags.

**Fig. 2:**
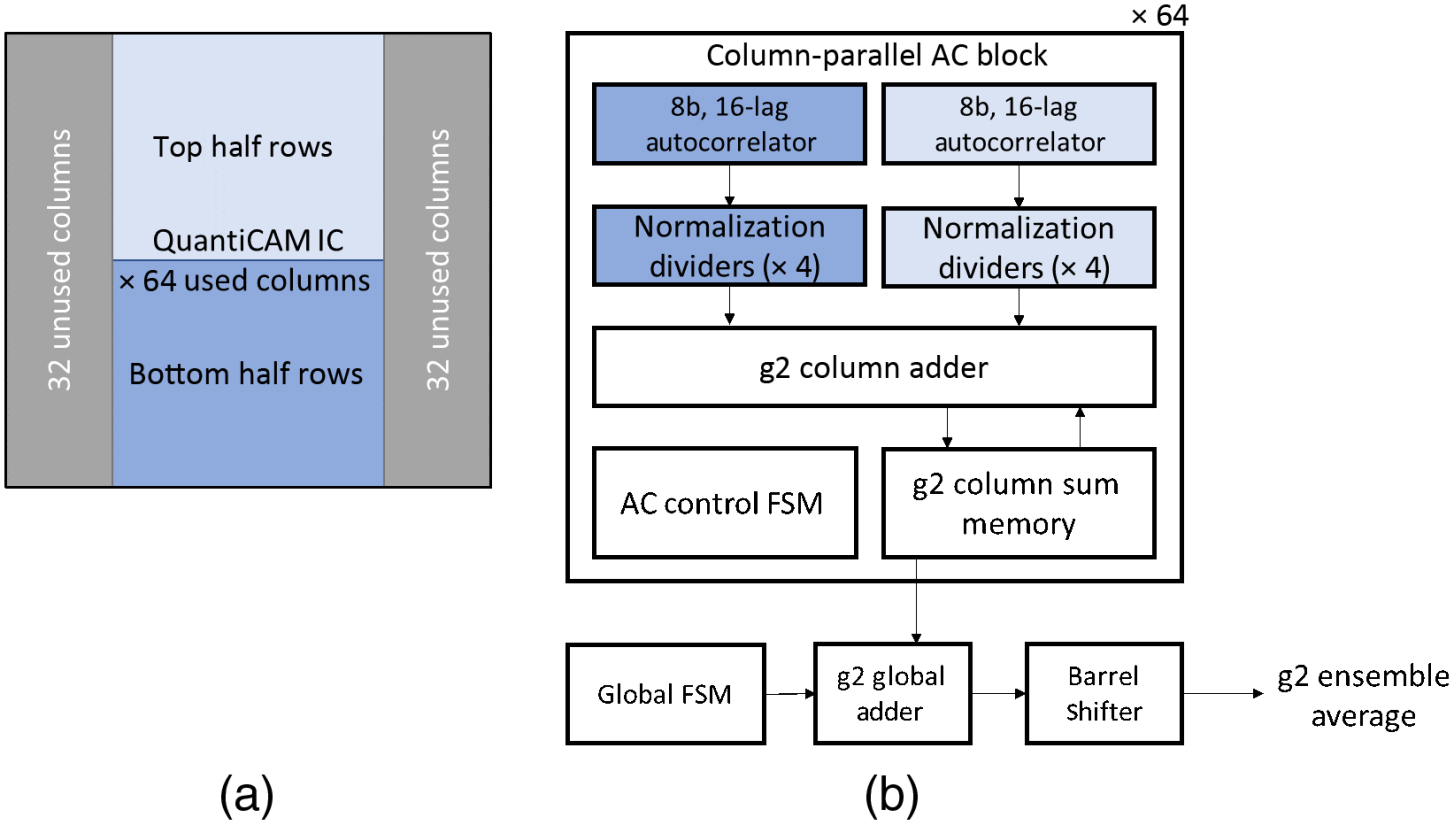
(a) Diagram of the column arrangement of the QuantICAM SPAD camera with the respect to the assignment to the column-parallel AC blocks. (b) The block diagram of each of the 64 column-parallel AC blocks.

The ACs were designed on FPGA to compute Eq. (3). The 128 ACs are grouped into pairs for a total of 64 blocks as shown in Fig. 2. Each of the 64 column-parallel AC blocks contains two sets of 8b, 16-lag ACs and two sets of 4 normalization dividers. Each set processes a half column of 96 pixels, one set for the top half of the array and one set for bottom half of the array. The column-parallel AC block is an instantiable module that can be replicated and easily connected to the rest of the surrounding logic. It is therefore simple to scale the number of column-parallel ACs from 64 to 128 blocks to serve the remaining unused columns of the SPAD array. Currently, this is a limitation of the chosen FPGA, but a larger off-the-shelf FPGAs would not impose such limit. The AC Control FSM controls the execution of the operations within a column-parallel AC block. The FSM is replicated for each column-parallel AC block as having it local to each block allowed better convergence of timing as opposed to routing control signal from a single shared FSM to all the 64 blocks. A g2 column adder and sum memory aggregate all per-pixel ACFs in a column. Finally, the global FSM, g2 global adder, and barrel shifter sum the g2 ACFs and take the average of columns into a single ensemble $g_{2}$ ACF for the array of 192×64  pixels.

**Fig. 3:**
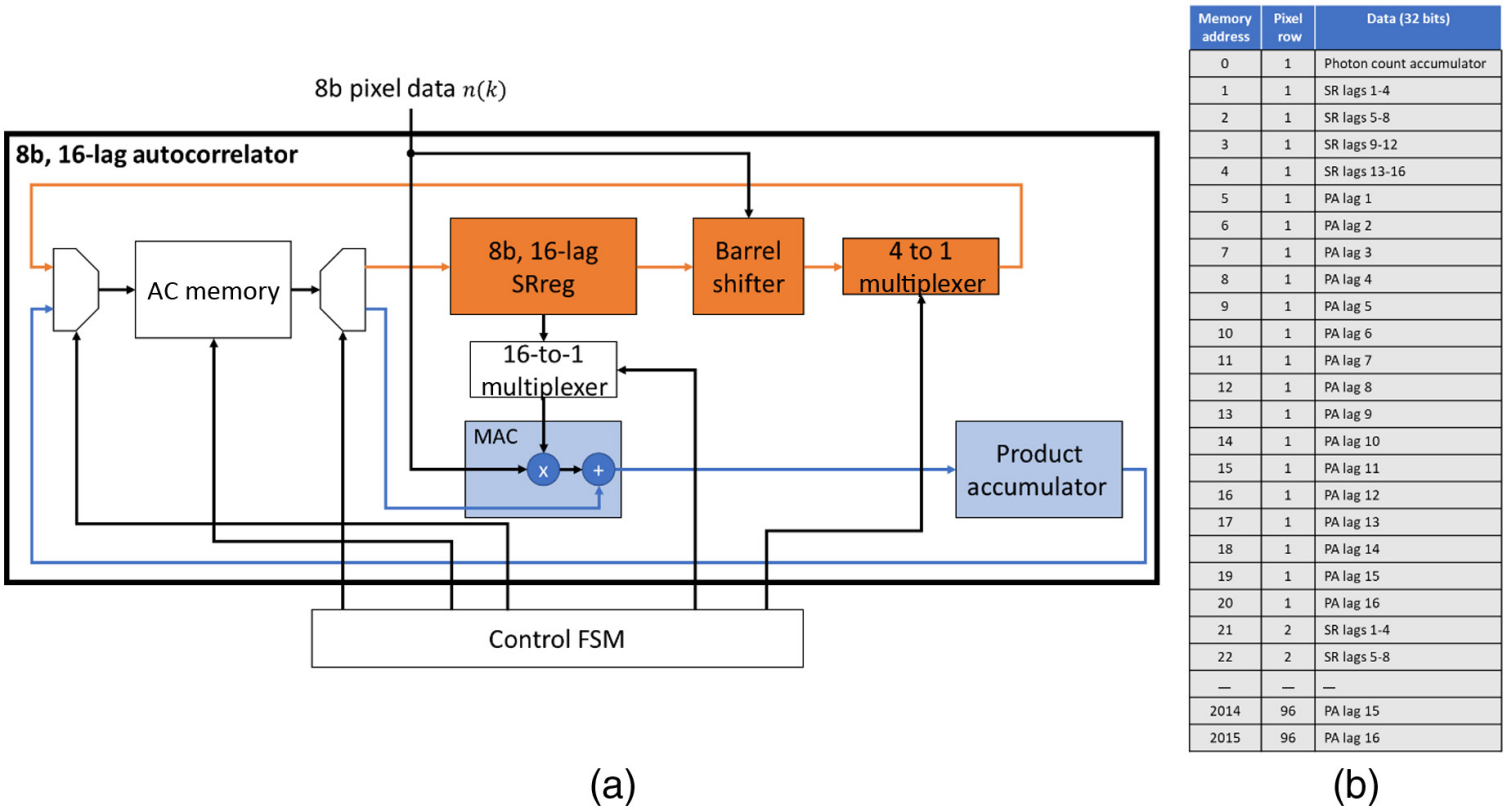
(a) Diagram of the blocks within an 8b, 16-lag AC. (b) Arrangement of data within the AC memory in each AC.

The block diagram of one of the 128 8b, 16-lag ACs is shown in Fig. 3(a). The AC is made up of an 8b, 16-element register (SRreg) to store the photon count of 16 consecutive frames. A barrel shifter is used to shift the SRreg by the new 8b frame count value n(k) and write the shifted SRreg back into the AC memory, acting as a virtual shift register for the 16 lag frames. A dual-port AC memory, as shown in Fig. 3(a), is used to store the value of the shift register and AC components for each lag over the integration period of N frames for all pixels in a half-column. In order to store the 8b shift register for 16 lags, four 32b memory addresses are required. The allocation of the $g_{2}$ components to the addresses in the AC memory is shown in Fig. 3(b).

Each of the 16 lags requires a multiplier to calculate the product of n(k) with each of the 16 previous frame counts n(k+τ). The products n(k)n(k+τ) are accumulated for each lag over an integration period of N frames and stored in the AC memory for each lag τ. These memory locations are known as product accumulators (PA) and there are 16 for each pixel. As multiply and accumulate cores (MACs) in the FPGA are limited, a single multiplier and integrator are shared among the 16 lags of each column AC. The FSM multiplexes the MAC for each lag.

To normalize each PA, the sum of the photons over all frames in an integration period is stored in a memory address for normalization at the end of the N frames. This AC component is known as the photon count accumulator (PCA). Each pixel requires 21 memory locations: 1 for the PCA, 4 for the shift register, and 16 for the PAs. In total to store the 96 pixels in a column, the AC memory is 32b by 2016 addresses.

The FSM controls the timing of the shift register update, multiplexing of the multiplication and accumulation of the all PAs and the integration of the PCA. The same FSM controls the reading and writing to the AC memory, computes and updates all memory locations for a pixel within a row-time to guarantee a frame rate of 26 kfps. The operations of the 8b, 16-lag AC at each new frame is summarized in Table 1. The AC takes 42 clock cycles of the 100-MHz system clock to update a pixel at each new frame.

**Table 1:** AC frame update operations.

Clock cycle	Operation description
1	• Read PCA value from AC memory
2	• Sum n(k) to read PCA value and write updated PCA value to AC memory
3	• Read SR lags 1 to 4 addresses and store SR lags in local register SRreg[0:3]
4	• Write to SR lags 1 to 4 addresses to update SR shifted with n(k) as first element of SR
5	• Read SR lags 5 to 8 addresses and store SR lags in local register SRreg[4:7]
6	• Write to SR lags 5 to 8 addresses to update SR shifted by one frame count
7	• Read SR lags 9 to 12 addresses and store SR lags in local register SRreg[8:11]
8	• Write to SR 9 to 12 addresses to update SR shifted by one frame count
9	• Read SR elements 13 to 16 addresses and store SR lags in local register SRreg[12:15]
10	• Write to SR 13 to 16 addresses to update SR shifted by one frame count
11	• Read PA for lag $f_{\tau }=1$
12	• MAC: ${\mathrm{PA}}_{\text{new}}(1)=\mathrm{SRreg}[0]\cdot n(k)+{\mathrm{PA}}_{\mathrm{prev}}$ and write MAC result in PA address for lag $f_{\tau }=1$
13-42	• Read PA for lag $f_{\tau }=2-16$ • MAC: ${\mathrm{PA}}_{\text{new}}(f_{\tau })=\mathrm{SRreg}[f_{\tau }-1]\cdot n(k)+{\mathrm{PA}}_{\mathrm{prev}}$ and write MAC result in PA address for lag $f_{\tau }=2-16$

To normalize the ACF, the PA values must be multiplied by the number of frames N in the integration period and divided by the PCA squared [see Eq. (3)]. At the end of the integration period, the contents of the AC memory are sent to the normalization dividers, which compute the square of the PCA and divide each lag PA by ${\mathrm{PCA}}_{2}$. The normalization operation of all pixels is completed in <2 frame times. The normalization starts as soon as the first pixel in the half-column has completed the last of the N frames and while the other pixels are finishing and updating the memory for the N’th frame. This ensures the normalization is completed before the pixels have to address the memory to start integrating again over a new N-frame integration period. As the division operation is a pipelined operation taking 20 clock cycles to compete for a single lag normalization, 4 dividers are used to compute 4 lags in parallel to fulfill the timing requirement of completing the normalization of all pixels in <2 frame times. This ensures that the normalization operation does not introduce a dead time in the acquisition to elaborate the data. The dual-port memory was chosen over the single-port option so that the integration and operations on the AC memory for the N’th frame could run concurrent to the dividers reading out and elaborating the pixel addresses that have already completed the AC operation for the N’th frame.

The normalized per-pixel ACFs can be optionally streamed out as they are being calculated by the column-parallel normalization dividers. This is a debug operation modality that allows the user to probe the individual per-pixel ACFs and monitor how these contribute to the SNR scaling. In the default modality, the eight dividers send the normalized per pixel ACFs to the g2 column adder [see Fig. 2(b)] which sums all the per-pixel ACFs of a column into the g2 column memory as each new pixel from the column is normalized. Once all pixels from a column are normalized and their ACFs summed and stored into the g2 column memory, the global g2 adder takes the sum of the ACFs from all columns. The final barrel shifter divides the result by 64 using a binary shift to normalize to the 64 columns. Division by the number of pixels in a column (192) and multiplication by the number of frames in the integration period N can be completed in software as it is a constant coefficient factor applied to the final ensemble $g_{2}$ result.

## 3 Results and Discussion

**Fig. 4:**
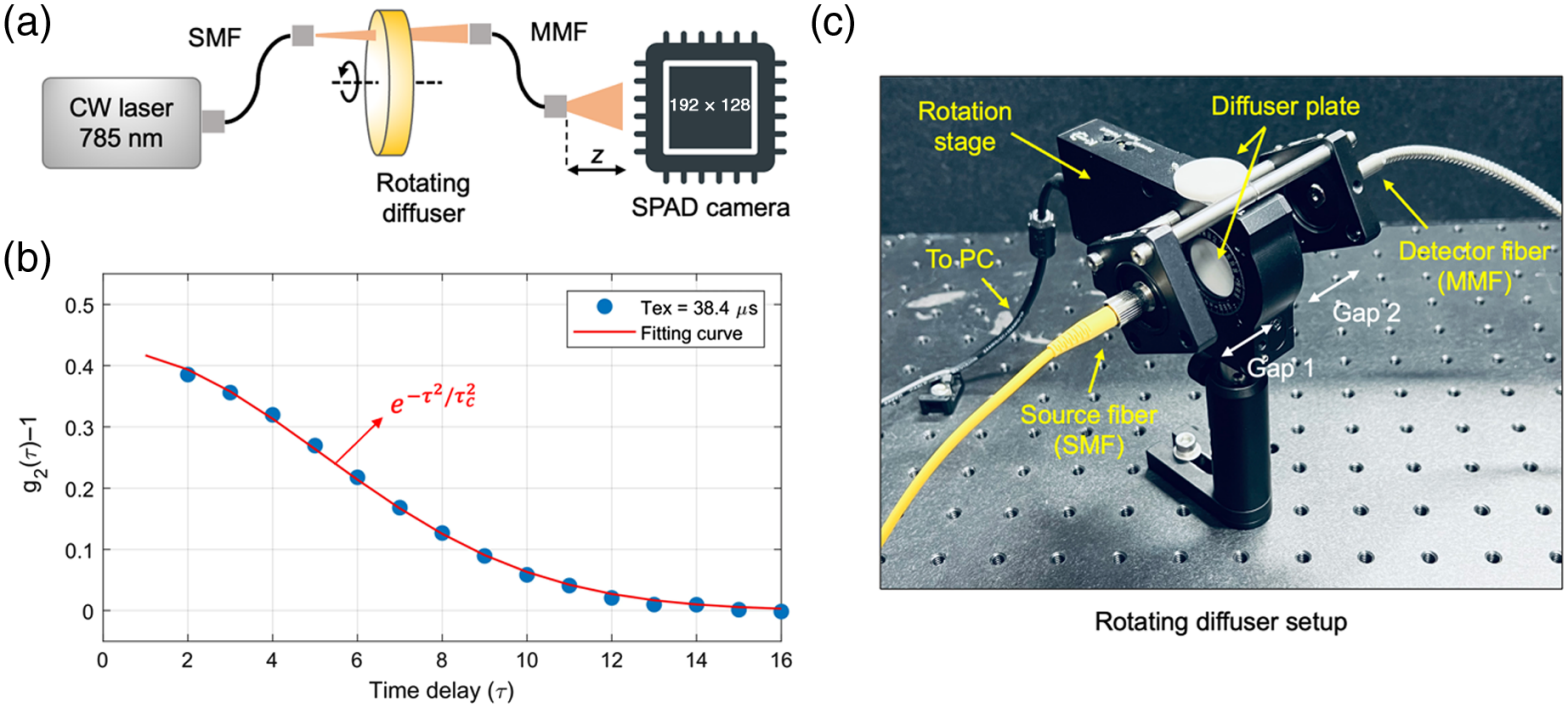
(a) Experimental setup for multispeckle DCS using a rotating diffuser phantom. (b) Ensemble averaged intensity ACF $g_{2}(\tau )$ of the photon counts from the rotating diffuser phantom. The measured $g_{2}(\tau )$ can be fitted well with $g_{2}(\tau )-1=\beta \;\exp(-\tau^{2}/\tau_{c}^{2})$, where β (=0.42) is the coherence factor, τ is the time delay in unit of the exposure time $T_{\mathrm{ex}}=38.4\;\mu s$, and $\tau_{c}$ (=7.2) is the correlation time. After converting the time delay unit, we obtained an estimated correlation time of $\tau_{c}=278\;\mu s$. (c) The speed of the motorized rotation stage (K10CR1, Thorlabs) was controlled by a user interface in PC. The diffuser plate (Biomimic™ optical phantom, INO) has the absorption coefficient $\mu_{a}=0.1\;{\mathrm{cm}}^{-1}$ and reduced scattering $\mu_{s^{\prime }}=13.4\;{\mathrm{cm}}^{-1}$, and it was cut into a cylindrical shape with dimension of 1 inch in diameter and 5-mm-thick. The source and detector fibers have their axes aligned, but offset from the center of the rotation by about 9 mm.

To demonstrate the capability of our large SPAD array with on-FPGA processing for increased SNR, we performed a rotating diffuser experiment. Figure 4(a) shows the experimental setup, consisting of a long coherence length (∼9 m) continuous wave laser emitting at 785 nm wavelength, a rotating diffuser plate, and a SPAD camera. Here we used the 192×128 SPAD camera (QuantICAM), but only half of the array was read out to FPGA as discussed above. The laser output was coupled to the phantom using a single-mode fiber. The diffused photons were collected by a multimode fiber (MMF) in a transmission geometry and coupled to the SPAD camera. The fiber-SPAD distance (z) was adjusted using a translating lens tube to calibrate the diameter of the speckles such that the average diameter of the speckles is at least twice larger than the SPAD active area (Nyquist).^12^ The SPAD camera consisted of 24,576 SPADs arranged in a 192×128 array, with a pixel pitch of 18.4  μm×9.2  μm and a 13% active fill factor. In comparison, the SPAD camera used in prior work was a 32×32 array, with a pixel pitch of 50  μm×50  μm and a 1.5% active fill factor.^6^^,^^7^

In the limit of low photon count rates (shot-noise limited regime), the SNR of ${\overset{¯}{g}}_{2}(\tau \to 0)$ is proportional to $N_{\mathrm{ph}}\times \sqrt{T_{\mathrm{int}}}\times \sqrt{M}$, where $N_{\mathrm{ph}}$ is the detected photon count rate, $T_{\mathrm{int}}$ is the integration time, and M is the number or pixels.^6^^,^^13^ Hence, in addition to the 24× larger pixel count, our SPAD camera offers slightly more than 8× larger active fill factor that allows a single MMF to couple the full-size SPAD array with independent speckle mode per pixel. To measure the speckle turnover time trace, we mounted the diffuser plate on a motorized rotation stage. The diffuser plate (Biomimic™ optical phantom, INO) has the absorption coefficient $\mu_{a}=0.1\;{\mathrm{cm}}^{-1}$ and reduced scattering coefficient $\mu_{s\prime }=13.4\;{\mathrm{cm}}^{-1}$. Figure 4(b) shows the measured intensity ACF $g_{2}(\tau )$ of the photon counts, where the time delay τ is in the unit of the exposure time $T_{\mathrm{ex}}=38.4\;\mu s$, with rolling shutter. The transmitted light was collected using MMF with 1000  μm core diameter.

**Fig. 5:**
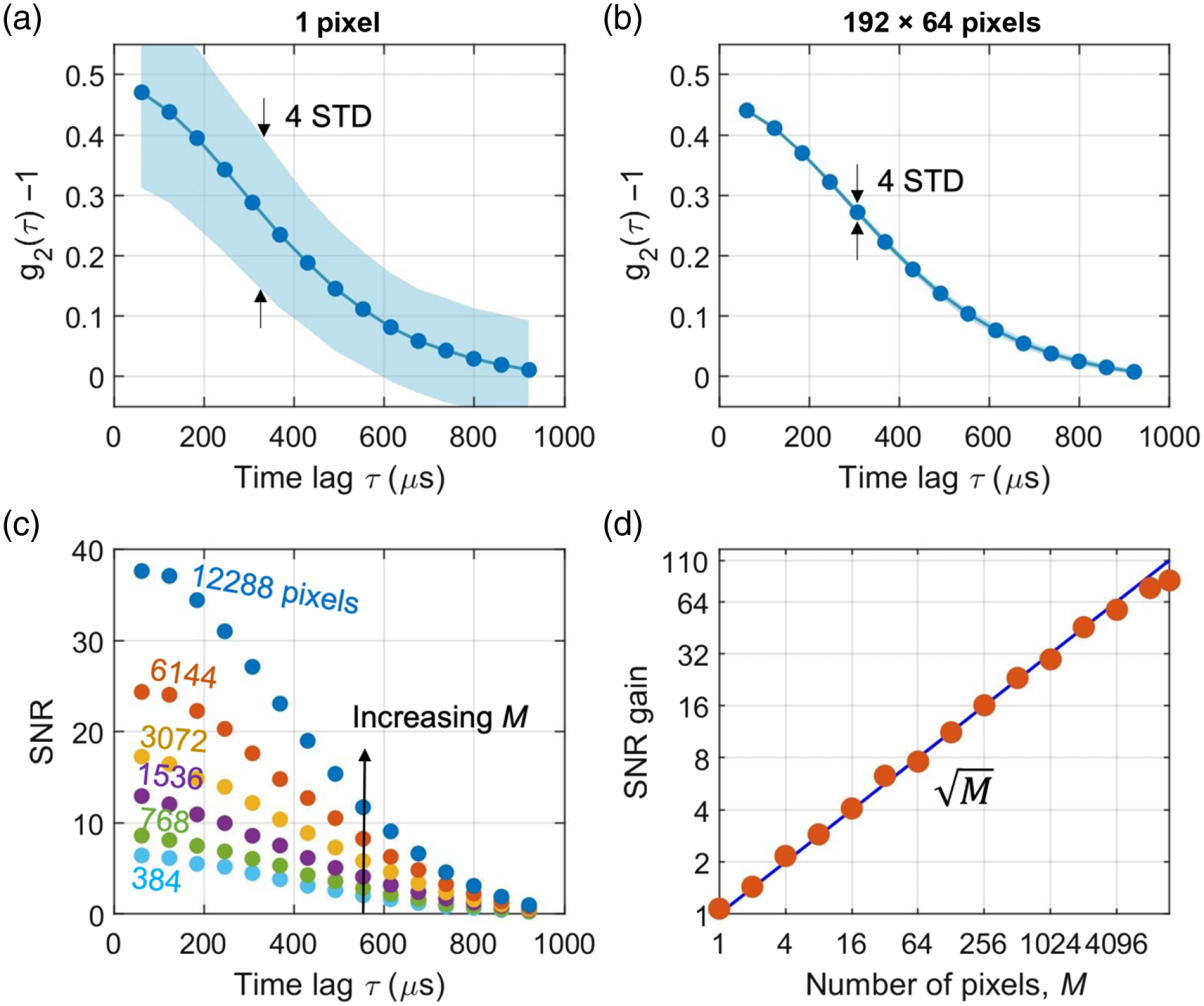
(a) Time statistics of the pixel intensity ACF $g_{2}^{i}(\tau )$. (b) Time statistics of the ensemble intensity ACF ${\overset{¯}{g}}_{2}(\tau )$. (c) SNR of ${\overset{¯}{g}}_{2}(\tau )$ for increasing size of the pixel ensemble M=1 to 12,288. (d) SNR gain of ${\overset{¯}{g}}_{2}(\tau =T_{\mathrm{ex}})$ as a function of the size of the pixel ensemble M=1 to 12,288. The exposure time was $T_{\mathrm{ex}}=61.44\;\mu s$.

To validate the SNR gain [defined as the ratio $\mathrm{SNR}[{\overset{¯}{g}}_{2}(\tau )]/\mathrm{SNR}[g_{2}^{i}(\tau )]$ for the first time bin], we compared the STD of the single-pixel $g_{2}^{i}$ and the ensemble averaged ${\overset{¯}{g}}_{2}$. Figure 5 shows separate rotating diffuser measurements at an angular speed of 12 deg/s, with SPAD exposure time $T_{\mathrm{ex}}=61.44\;\mu s$. The solid line in Fig. 5(a) ($g_{2}^{i}$) and 5b (${\overset{¯}{g}}_{2}$) corresponds to the mean value of $g_{2}(\tau )$. The shade corresponds to ±2 STD with respect to the mean value. The STD of ${\overset{¯}{g}}_{2}(\tau =T_{\mathrm{ex}})$ [Fig. 3(b)] is about 110 times smaller than that of $g_{2}^{i}(\tau =T_{\mathrm{ex}})$ [Fig. 3(a)], which is much smaller than the size of the symbols in the plot. Figure 5(c) shows the SNR of ${\overset{¯}{g}}_{2}(\tau )$ at increasing size of the pixel ensemble M of up to 12,288. Averaging over a larger ensemble of pixels leads to higher SNR. Figure 5(d) shows the SNR gain of ${\overset{¯}{g}}_{2}(\tau =T_{\mathrm{ex}})$ as a function of the size of the pixel ensemble M of up to 12,288, normalized to that of the single-pixel. The measured SNR gain (solid circles) increases as the square root of the ensemble size (solid line), as predicted by the multispeckle DCS model.^6^

## 4 Conclusion

We have demonstrated an SNR gain of 110 with respect to the single-pixel multispeckle DCS using half of the 192×128 SPAD array, with pixel active fill factor of 13%. This is an order of magnitude larger pixel count than the 32×32 SPAD array with fill factor of 1.5% in prior works.^6^^,^^7^ In addition to the large pixel count, we have also demonstrated a real-time data compression scheme implemented in the readout of the SPAD camera by calculating the ensemble $g_{2}$ ACF entirely on FPGA, showing an improvement of over an order of magnitude in the number of parallel channels that can be processed in real time. Shifting the $g_{2}$ calculations from a high-performance workstation computer to FPGA has enabled the running of real-time multispeckle DCS measurements using a standard PC, e.g., a laptop. This effort should help democratize the use of large-pixel-count SPAD cameras for multispeckle DCS in broad biomedical optics research. Although the FPGA-embedded processing plays a crucial role to jump start large array multispeckle DCS research due to its reconfigurability as we explore different operation modes (e.g., photon-counting, DCS, and time-domain DCS), our complete on-FPGA processing chain demonstrates that the digital circuit resources required are compatible with embedded on-chip-based processing. Thus we envision that ultimately real-time data compression is achievable in an advanced computational SPAD camera based with integrated focal-plane autocorrelators.

Finally, recent theoretical investigation by Cheng et al.^14^ suggested that to be sensitive to local, subsecond latency hemodynamic response of the cerebral cortex at ∼15  mm beneath the scalp surface, we need a shorter exposure time that can resolve the ACF with a time constant of the order of tens of μs and a larger pixel count of the order of $10^{4}$ to $10^{6}\text{ pixels}$. Although our results with on-FPGA processing offer great promise for multispeckle DCS, currently the shortest exposure time of our SPAD camera is limited to $T_{\mathrm{ex}}=38.4\;\mu s$ and the photon detection probability of the pixel is ∼8% at an excess bias of 1.7 V and wavelength of 785 nm.^11^^,^^15^ Despite this exposure time limitation, we believe that the QuantICAM DCS system can benefit real-time applications, such as neuroscience for smaller animals with skull/scalp thickness much smaller than that of humans which leads to longer correlation time, thus can be measured using longer exposure time from QuantICAM SPAD camera. Other examples include measurements on human extracerebral blood flow and human peripheral blood flow (e.g., wrist) for bedside health monitoring. Another example includes regression from extracerebral blood flow using short source–detector separation that leads to correlation time of the order of tens of μs.

Intense research effort in SPAD camera development is currently ongoing to improve SPAD camera capabilities toward even larger pixel count ($∼10^{6}$), shorter exposure time (sub-μs), and higher detection probability.^16^^–^^18^ Soon, we should expect high-performance SPAD cameras with FPGA-embedded or even on-chip computing that could surpass the multispeckle DCS requirements for noninvasive detection of local brain activation.

## Code, Data, and Materials Availability

The data and code (FPGA Verilog RTL and MATLAB software for data elaboration) that support the findings of this study are available from the corresponding author on reasonable request.
